# Supplementary material for: The Systems Biology Research Tool: evolvable open-source software
Source: BMC Syst Biol. 2008 Jun 29;2:55. doi: 10.1186/1752-0509-2-55 (PMC2446383; doi:10.1186/1752-0509-2-55)
Supplement: Additional file 1 — SBRT Archive. An archive of the current version of the Systems Biology Research Tool. [file 1752-0509-2-55-S1.zip › sbrt-1.4.0/doc/developers_guide/api/sbrt/ext/cplex/CplexSolver.html]

CplexSolver


|  |  |  |
| --- | --- | --- |
| **Overview** \| **Package** \| **Class** \| **Use** \| **Tree** \| **Deprecated** \| **Index** \| **Help** | |  |
| PREV CLASS   NEXT CLASS | **FRAMES**    **NO FRAMES**     **All Classes** |  |
| SUMMARY: NESTED \| FIELD \| CONSTR \| METHOD | DETAIL: FIELD \| CONSTR \| METHOD |  |


---


## sbrt.ext.cplex Class CplexSolver<R,C>

```
java.lang.Object
  sbrt.ext.cplex.CplexSolver<R,C>
```

**Type Parameters:**: `R` - the row variable type.: `C` - the column variable type.

**All Implemented Interfaces:**: LinearProgramSolver<R,C>, ProgramSolver<R,C>, QuadraticProgramSolver<R,C>

---

``` public final class CplexSolver<R,C> extends java.lang.Object implements LinearProgramSolver<R,C>, QuadraticProgramSolver<R,C> ```

This class is a wrapper for the CPLEX package from ILOG.

**Author:**
:   This class was written and documented by Jeremiah Wright
    while in the Wagner lab.

---

**Constructor Summary**

`CplexSolver()`
  
          Constructs a new CPLEX program solver.


| **Method Summary** | |
| --- | --- |
| `void` | `clear()`             Clears all information contained in this program solver. |
| `double` | `getObjectiveValue()`             Returns the most recently computed objective value. |
| `UnmodifiableMap<C,java.lang.Double>` | `getSolutionVector()`             Returns the most recently computed solution vector. |
| `ProgramSolverStatus` | `getStatus()`             Returns the status of the most recently attempted optimization. |
| `boolean` | `isColumnVar(java.lang.Object obj)`             Indicates if the provided object is a column variable in the matrix of this program solver. |
| `boolean` | `isRowVar(java.lang.Object obj)`             Indicates if the provided object is a row variable in the matrix of this program solver. |
| `boolean` | `optimize()`             Computes the optimal value of the current objective function in the direction of the current optimization sense. |
| `<T extends C> void` | `setColumnConstraint(T column, Interval c)`             Sets the constraint on the provided column variable. |
| `void` | `setColumnConstraints(java.util.Map<? extends C,Interval> constraints)`             Sets the constraints for the provided column variables. |
| `void` | `setConstraint(LinearComb<? extends C> linComb, Interval c)`             Sets a constraint on the provided mathematical expression of column variables. |
| `void` | `setConstraint(MathExpr<? extends C> expression, Interval c)`             Sets a constraint on the provided mathematical expression of column variables. |
| `void` | `setConstraint(QuadraticExpr<? extends C> expr, Interval c)`             Sets a constraint on the provided mathematical expression of column variables. |
| `void` | `setConstraints(java.util.Map<? extends MathExpr<? extends C>,Interval> exprConstraints)`             Sets constraints on the provided mathematical expressions of column variables. |
| `<S extends R,T extends C> void` | `setMatrixElement(S row, T column, double value)`             Sets the element of the program matrix at the provided row and column variables to the one provided. |
| `void` | `setObjectiveFunction(LinearComb<? extends C> objective)`             Sets the objective function to the provided mathematical expression of column variables. |
| `void` | `setObjectiveFunction(LinearComb<? extends C> objective, OptimizationSense sense)`             Sets the objective function and optimization sense to the provided values. |
| `void` | `setObjectiveFunction(MathExpr<? extends C> objective)`             Sets the objective function to the provided mathematical expression of column variables. |
| `void` | `setObjectiveFunction(MathExpr<? extends C> objective, OptimizationSense sense)`             Sets the objective function and optimization sense to the provided values. |
| `void` | `setObjectiveFunction(QuadraticExpr<? extends C> objective)`             Not yet implemented. |
| `void` | `setObjectiveFunction(QuadraticExpr<? extends C> objective, OptimizationSense sense)`             Sets the objective function and optimization sense to the provided values. |
| `void` | `setOptimizationSense(OptimizationSense sense)`             Sets the optimization sense. |
| `void` | `setParamFile(java.lang.String prmFileName)`             Reads the program solver parameter file with the provided name. |
| `<T extends R> void` | `setRowConstraint(T row, Interval c)`             Sets the constraint for the provided row variable. |
| `void` | `setRowConstraints(java.util.Map<? extends R,Interval> rowConstraints)`             Sets the constraints for the provided row variables. |
| `java.lang.String` | `toString()`             Returns the name of this program solver. |
| `void` | `writeProblem(java.lang.String fileName)`             Writes the CPLEX model to a file with the provided name. |

**Methods inherited from class java.lang.Object**

`clone, equals, finalize, getClass, hashCode, notify, notifyAll, wait, wait, wait`

**Constructor Detail**

### CplexSolver

```
public CplexSolver()
```

:   Constructs a new CPLEX program solver.


**Method Detail**

### clear

```
public void clear()
```

:   Clears all information contained in this program solver. Calling
    this method restores this program solver to its original, pristine
    state.

    :   **Specified by:**: `clear` in interface `ProgramSolver<R,C>`

---


### setMatrixElement

```
public <S extends R,T extends C> void setMatrixElement(S row,
                                                       T column,
                                                       double value)
```

:   Sets the element of the program matrix at the provided row and
    column variables to the one provided.

    :   **Specified by:**: `setMatrixElement` in interface `ProgramSolver<R,C>`
    :   **Type Parameters:**: `S` - a type extending the primary row variable type.: `T` - a type extending the primary column variable type. **Parameters:**: `row` - the row variable.: `column` - the column variable.: `value` - the double precision element value.

---


### setConstraint

```
public void setConstraint(MathExpr<? extends C> expression,
                          Interval c)
```

:   Sets a constraint on the provided mathematical expression of
    column variables.

    :   **Specified by:**: `setConstraint` in interface `ProgramSolver<R,C>`
    :   **Parameters:**: `expression` - a mathematical expression of column variables for which the constraint is set.: `c` - the constraint.

---


### setConstraints

```
public void setConstraints(java.util.Map<? extends MathExpr<? extends C>,Interval> exprConstraints)
```

:   Sets constraints on the provided mathematical expressions of
    column variables.

    :   **Specified by:**: `setConstraints` in interface `ProgramSolver<R,C>`
    :   **Parameters:**: `exprConstraints` - mathematical expressions of column variables mapped to their corresponding constraint.

---


### setConstraint

```
public void setConstraint(LinearComb<? extends C> linComb,
                          Interval c)
```

:   Sets a constraint on the provided mathematical expression of
    column variables.

    :   **Specified by:**: `setConstraint` in interface `LinearProgramSolver<R,C>`
    :   **Parameters:**: `linComb` - a mathematical expression of column variables for which the constraint is set.: `c` - the constraint.

---


### setConstraint

```
public void setConstraint(QuadraticExpr<? extends C> expr,
                          Interval c)
```

:   Sets a constraint on the provided mathematical expression of
    column variables.

    :   **Specified by:**: `setConstraint` in interface `QuadraticProgramSolver<R,C>`
    :   **Parameters:**: `expr` - a mathematical expression of column variables for which the constraint is set.: `c` - the constraint.

---


### setColumnConstraint

```
public <T extends C> void setColumnConstraint(T column,
                                              Interval c)
```

:   Sets the constraint on the provided column variable.

    :   **Specified by:**: `setColumnConstraint` in interface `ProgramSolver<R,C>`
    :   **Type Parameters:**: `T` - a type extending the primary column variable type. **Parameters:**: `column` - the column variable for which the constraint is set.: `c` - the constraint.

---


### setColumnConstraints

```
public void setColumnConstraints(java.util.Map<? extends C,Interval> constraints)
```

:   Sets the constraints for the provided column variables.

    :   **Specified by:**: `setColumnConstraints` in interface `ProgramSolver<R,C>`
    :   **Parameters:**: `constraints` - column variables mapped to their corresponding constraint.

---


### setRowConstraint

```
public <T extends R> void setRowConstraint(T row,
                                           Interval c)
```

:   Sets the constraint for the provided row variable.

    :   **Specified by:**: `setRowConstraint` in interface `ProgramSolver<R,C>`
    :   **Type Parameters:**: `T` - a type extending the primary row variable type. **Parameters:**: `row` - the row variable for which the constraint is set.: `c` - the constraint.

---


### setRowConstraints

```
public void setRowConstraints(java.util.Map<? extends R,Interval> rowConstraints)
```

:   Sets the constraints for the provided row variables.

    :   **Specified by:**: `setRowConstraints` in interface `ProgramSolver<R,C>`
    :   **Parameters:**: `rowConstraints` - row variables mapped to their corresponding constraint.

---


### setObjectiveFunction

```
public void setObjectiveFunction(MathExpr<? extends C> objective,
                                 OptimizationSense sense)
```

:   Sets the objective function and optimization sense to the provided
    values.

    :   **Specified by:**: `setObjectiveFunction` in interface `ProgramSolver<R,C>`
    :   **Parameters:**: `objective` - a mathematical expression of column variables from this program solver.: `sense` - the optimization sense. **See Also:**: `setObjectiveFunction(MathExpr)`, `setOptimizationSense(OptimizationSense)`

---


### setObjectiveFunction

```
public void setObjectiveFunction(LinearComb<? extends C> objective,
                                 OptimizationSense sense)
```

:   Sets the objective function and optimization sense to the provided
    values.

    :   **Specified by:**: `setObjectiveFunction` in interface `LinearProgramSolver<R,C>`
    :   **Parameters:**: `objective` - a mathematical expression of column variables from this program solver.: `sense` - the optimization sense. **See Also:**: `setObjectiveFunction(MathExpr)`, `setOptimizationSense(OptimizationSense)`

---


### setObjectiveFunction

```
public void setObjectiveFunction(QuadraticExpr<? extends C> objective,
                                 OptimizationSense sense)
```

:   Sets the objective function and optimization sense to the provided
    values.

    :   **Specified by:**: `setObjectiveFunction` in interface `QuadraticProgramSolver<R,C>`
    :   **Parameters:**: `objective` - a mathematical expression of column variables from this program solver.: `sense` - the optimization sense. **See Also:**: `setObjectiveFunction(MathExpr)`, `setOptimizationSense(OptimizationSense)`

---


### setObjectiveFunction

```
public void setObjectiveFunction(MathExpr<? extends C> objective)
```

:   Sets the objective function to the provided mathematical expression of
    column variables.

    :   **Specified by:**: `setObjectiveFunction` in interface `ProgramSolver<R,C>`
    :   **Parameters:**: `objective` - a mathematical expression of column variables from this program solver.

---


### setObjectiveFunction

```
public void setObjectiveFunction(LinearComb<? extends C> objective)
```

:   Sets the objective function to the provided mathematical expression of
    column variables.

    :   **Specified by:**: `setObjectiveFunction` in interface `LinearProgramSolver<R,C>`
    :   **Parameters:**: `objective` - a mathematical expression of column variables from this program solver.

---


### setObjectiveFunction

```
public void setObjectiveFunction(QuadraticExpr<? extends C> objective)
```

:   Not yet implemented.

    :   **Specified by:**: `setObjectiveFunction` in interface `QuadraticProgramSolver<R,C>`
    :   **Parameters:**: `objective` - a mathematical expression of column variables from this program solver. **Throws:**: `java.lang.UnsupportedOperationException`

---


### setOptimizationSense

```
public void setOptimizationSense(OptimizationSense sense)
```

:   Sets the optimization sense.

    :   **Specified by:**: `setOptimizationSense` in interface `ProgramSolver<R,C>`
    :   **Parameters:**: `sense` - the optimization sense.

---


### optimize

```
public boolean optimize()
```

:   Computes the optimal value of the current objective function in the
    direction of the current optimization sense.

    :   **Specified by:**: `optimize` in interface `ProgramSolver<R,C>`
    :   **Returns:**: `true` if an optimal solution was found; `false` otherwise. **See Also:**: `getObjectiveValue()`, `getSolutionVector()`

---


### getStatus

```
public ProgramSolverStatus getStatus()
```

:   Returns the status of the most recently attempted optimization.

    :   **Specified by:**: `getStatus` in interface `ProgramSolver<R,C>`
    :   **Returns:**: the status of the most recently attempted optimization. **See Also:**: `optimize()`

---


### getObjectiveValue

```
public double getObjectiveValue()
```

:   Returns the most recently computed objective value.

    :   **Specified by:**: `getObjectiveValue` in interface `ProgramSolver<R,C>`
    :   **Returns:**: the most recently computed objective value. **See Also:**: `optimize()`

---


### getSolutionVector

```
public UnmodifiableMap<C,java.lang.Double> getSolutionVector()
```

:   Returns the most recently computed solution vector.

    :   **Specified by:**: `getSolutionVector` in interface `ProgramSolver<R,C>`
    :   **Returns:**: column variables mapped to their corresponding numerical values. **See Also:**: `optimize()`

---


### writeProblem

```
public void writeProblem(java.lang.String fileName)
```

:   Writes the CPLEX model to a file with the provided name.

    :   **Parameters:**: `fileName` - the name of the file to be written.

---


### toString

```
public java.lang.String toString()
```

:   Returns the name of this program solver.

    :   **Overrides:**: `toString` in class `java.lang.Object`
    :   **Returns:**: the name of this program solver.

---


### setParamFile

```
public void setParamFile(java.lang.String prmFileName)
```

:   Reads the program solver parameter file with the provided name.
    This file must be in PRM format. See the CPLEX documentation for further
    details.

    :   **Specified by:**: `setParamFile` in interface `ProgramSolver<R,C>`
    :   **Parameters:**: `prmFileName` - the name of the PRM file containing parameter values.

---


### isColumnVar

```
public boolean isColumnVar(java.lang.Object obj)
```

:   Indicates if the provided object is a column variable in the matrix of
    this program solver.

    :   **Specified by:**: `isColumnVar` in interface `ProgramSolver<R,C>`
    :   **Parameters:**: `obj` - the potential column variable. **Returns:**: `true` if the provided object is a column variable in the matrix of this program solver; `false` otherwise.

---


### isRowVar

```
public boolean isRowVar(java.lang.Object obj)
```

:   Indicates if the provided object is a row variable in the matrix of
    this program solver.

    :   **Specified by:**: `isRowVar` in interface `ProgramSolver<R,C>`
    :   **Parameters:**: `obj` - the potential row variable. **Returns:**: `true` if the provided object is a row variable in the matrix of this program solver; `false` otherwise.


---


|  |  |  |
| --- | --- | --- |
| **Overview** \| **Package** \| **Class** \| **Use** \| **Tree** \| **Deprecated** \| **Index** \| **Help** | |  |
| PREV CLASS   NEXT CLASS | **FRAMES**    **NO FRAMES**     **All Classes** |  |
| SUMMARY: NESTED \| FIELD \| CONSTR \| METHOD | DETAIL: FIELD \| CONSTR \| METHOD |  |


---
